# Pain-Administrable Neuron Electrode with Wireless Energy Transmission: Architecture Design and Prototyping

**DOI:** 10.3390/mi12040356

**Published:** 2021-03-25

**Authors:** Chin-Yu Lin, Li-Chi Chang, Jyh-Chern Chen, Meng-Sheng Chen, Hsun Yu, Mei-Chih Wang

**Affiliations:** 1 Institute of New Drug Development, China Medical University, Taichung 40402, Taiwan; 2 Master Program for Biomedical Engineering, Collage of Biomedical Engineering, China Medical University, Taichung 40402, Taiwan; 3 Graduate Institute of Communication Engineering, National Taiwan University, Taipei City 10617, Taiwan; D02942003@ntu.edu.tw; 4 ACE Biotek Co., Ltd., Hsinchu 30261, Taiwan; 5 Electronic & Optoelectronic System Research Laboratories, Industrial Technology Research Institute, Hsinchu 31057, Taiwan; mason@itri.org.tw (M.-S.C.); syunyu@itri.org.tw (H.Y.); 6 Biomedical Technology & Device Research Laboratories, Industrial Technology Research Institute, Hsinchu 31057, Taiwan

**Keywords:** wireless energy transmission, spinal cord stimulation, neural stimulatory electrode, PRF stimulatory electrode, circuits design for wireless energy transmission

## Abstract

Back pain resulted from spine disorders reaches 60–80% prevalence in humans, which seriously influences life quality and retards economic production. Conventional electrical pain relief therapy uses radiofrequency to generate a high temperature of 70–85 °C on the electrode tip to destroy the neural transmission and stop the pain. However, due to the larger area of stimulation, eliciting significant side effects, such as paralysis, contraction, and a slightly uncomfortable feeling, our study aimed to design a tiny and stretchable neural stimulatory electrode that could be precisely anchored adjacent to the dorsal root ganglion which needs therapy and properly interfere with the sensory neural transmission. We also designed a subcutaneously implantable wireless power transmission (WPT) device to drive the neural stimulatory electrode. Through the study, we elaborated the design concept and clinical problems, and achieved: (1) the architecture design and simulation of the transdermal wireless power transferred device, (2) a wrap-able pulsed radiofrequency (PRF) stimulatory electrode, (3) an insulation packaging design of the titanium protection box. The feasibility study and hands-on prototype were also carried out.

## 1. Introduction

The neck or back pain resulting from spine disorders, including acute back pain and chronic lower back pain that continues for several months, reaches 60–80% prevalence in humans. Although these kinds of pain would not immediately affect human life, they do seriously influence life quality and retard economic production. Current strategies used for pain control clinically include oral painkillers, Nonsteroidal anti-inflammatory drugs (NSAID), physical therapy, transdermal electrical stimulators, invasive pulsed radiofrequency (PRF) stimulators, implantable spinal cord electrical stimulators (SCS), and intraspinal cord injections [1,2,3,4]. A population of over 75 million in U.S. are suffering from chronic neuron disease-induced pain, and the costs of therapeutics and medical treatments have reached 2.3 billion with a year-on-year (YOY) of 12.1% [5,6].

Pain feeling transmission was impeded to achieve pain relief by the electric field effect released from the implanted PRF neural stimulatory device which stimulated neurons in the transmission pathway. A therapeutic temperature lower than 42 °C of the PRF neural stimulatory device would possess good pain relief and lower the risk of tissue heating damage and nearby neural capability loss. However, the therapeutic duration of the PRF stimulatory device is limited, so patients with chronic back pain need to repeat the identical therapeutic operation 3–6 months later [7,8]. Besides, the PRF stimulatory device is single-use and expensive, resulting in the patient’s heavy medical burden. Therefore, the development of the wireless energy transferred PRF neural stimulatory device will possess a high impact on the clinical application as well as largely reduce the risk of patient and health care personnel from exposure to radiology during therapeutic operations [9,10].

Generally, neuron stimulatory electrodes can be classified into the rod-shaped type applied on the brain cortex implantation and the patch type for in vivo adhesion through surgery, which all should possess biocompatibility and non-toxicity to humans in compliance with the Food and Drug Administration (FDA) regulation for the medical device. The patched electrode generally adheres onto the dura layer in the spinal cord through microsurgery, however, the drawback is that it is difficult to control the stimulatory site precisely and the stimulus will affect a large area. Therefore, the patch-type stimulation would impact the pain transmission, but also might impede the motor neuron transmission [11].

In this study, we would like to design and manufacture a novel PRF neuron stimulatory electrode with stretchable anchors to address pain relief. Through microsurgery, the PRF electrode could penetrate the spinal dura guided by a stainless needle to reach and precisely anchor on the soft tissue around the dorsal root ganglion, then elicit the therapeutic stimulus and block pain feeling. High grade implantable medical devices is an emerging composite industry combined with medicine, chemistry, biomaterials, mechanics, and photo-electronics, creating tremendous research potential and economic value. However, biomedical products are distinct from conventional consumables; they are stringently regulated by the FDA, guaranteeing their safety concerns. Therefore, developing high-grade implantable medical devices would establish several mutual core platforms, such as wireless energy transmission, durable and flexible electrodes, and biocompatible insulation, which could be applied for many distinct implant developments and industrial chain promotion.

## 2. Materials and Methods

### 2.1. Wire Loops, Architecture Design, and Simulation of Wireless Power Transmission

The wireless power transmission (WPT) system in this study was designed using a resonant coupling technique for neuron stimulatory electrodes. Mathematical analysis and simulations of various wire loop designs were carried out by two electromagnetic (EM)-simulation tools and a calculating software, ANSYS HFSS (Version 15.0, ANSYS, Inc., Canonsburg, PA, USA), CST STUDIO SUITE^®^ (CST Studio Suite 2019, Dassault Systèmes, Inc., Waltham, MA, USA), and PathWave ADS circuit simulator (W3600B, Keysight Technologies, Inc., Santa Rosa, CA, USA), respectively. ANSYS HFSS was used to set various parameters for modeling the transmitter and the receiver coupling-wire loops to calculate the transmission efficiency, such as wire loops size, shape, quantity factor of coils, the line width of wires, self-inductance, mutual inductance, coupling coefficient, and magnetic flux magnitude. CST STUDIO SUITE^®^ was adopted for designing the resonate dual-loop method for enhancing the WPT distance. PathWave ADS was used to design the matching network of WPT and also calculate the power attenuation and efficiency of the proposed dual-loop design.

The efficiency attenuation between the wireless power transmitter and the receiver was simulated through the following equation:

(1) $$\mathrm{Attenuation}\;\mathrm{decibels}\;\left(\mathrm{dB}\right)=10\times \log_{10}\left(\frac{{\mathrm{Power}}_{\mathrm{input}}}{{\mathrm{Power}}_{\mathrm{output}}}\right)$$

(2) $$\mathrm{Efficiency}\;\left(\%\right)=10^{\frac{\mathrm{attenuation}}{10}}\times 100\%$$

### 2.2. Neuron Stimulatory Electrode Design and Prototyping

To design a PRF neuron stimulatory electrode through microsurgery for implantation, we designed a flexible electrode, which could be wrapped in a guiding needle, penetrating the spinal dura, reaching and anchoring on soft tissue around the dorsal root ganglion which needed therapeutic stimuli. According to human anatomy, the dorsal root ganglion, with a size approximately 3–6 mm in diameter and 4–11 mm in length, is surrounded with soft tissues. The electrode would be delivered by a needle, which might not directly reach the target “dorsal root ganglion”. Therefore, we designed a protective cap on the tip of the electrode with expandable anchors. Distinct from conventional electrodes to release electricity on the tip and heat the tissues orthotopically, the PRF electrode impeded neural transmission through the specified electric signal generated from the microcontroller, which provided power by the wireless power transfer design in a different frequency. Therefore, the electrode wire could be designed on the anchors. For prototyping, we selected polyimide as the base material to coat metal electrodes for biocompatibility. A 250 × 6 × 1 mm supporting glass plate substrate was marked by mechanical engraving. Subsequently, an about 250 μm-thick layer of polyimide BPDA/PDA (derived from 3,3′,4,4′-biphenyltetracarboxylic dianhydride (BPDA) and *p*-phenylendiamine (PDA)) was applied onto the front side of the glass plate as a liquid precursor by spin coating and cured to polyimide (PI 2611, HD MicroSystems, Parlin, NJ, USA). A 1 μm layer of titanium was applied on the polyimide layer and a 5 μm layer of platinum was applied on the titanium layer by magnetron sputtering sequentially, followed by a final 1 μm titanium layer, yielding a titanium/platinum/titanium thin film stack.

**Figure 1:**
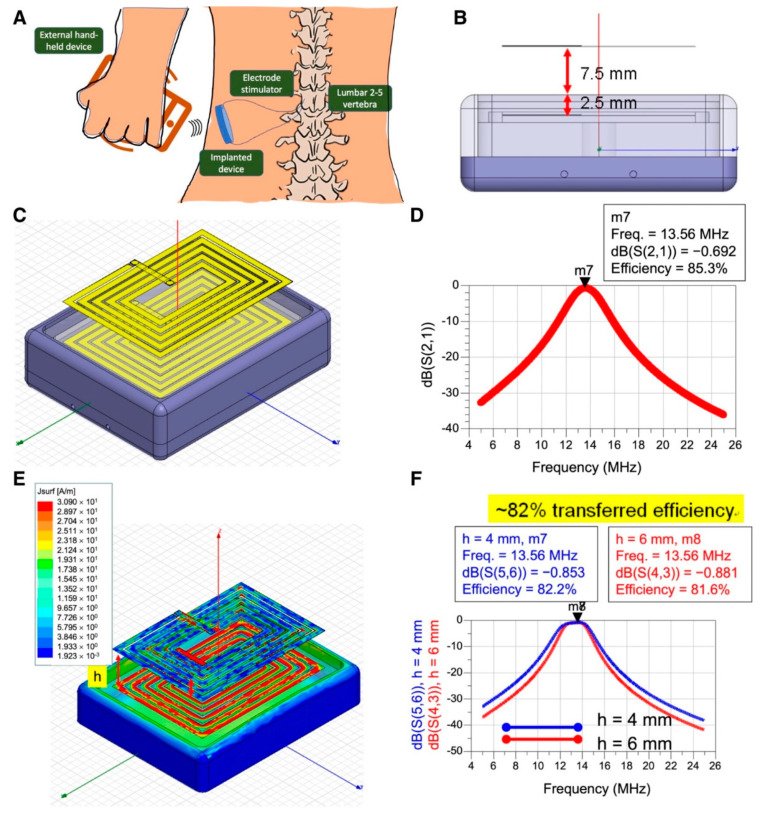
Rectangular-shaped wireless power transmission box and wire design. (**A**) Illustration of system model construction used in this 3D simulation for wireless energy transmission. (**B**) Transversal view of the rectangular device. The plane of the wires is placed 2.5 mm under the case cover, and the subcutaneous implantation depth is 7.5 mm. (**C**) Architecture design of cortex wires of the transmitter and receiver. (**D**) Simulated attenuation of wireless power transmission in various frequencies. (**E**) Current distribution in cortex wires in the receiver, and the distance between both cortex wires is set 10 mm. (**F**) Simulated attenuation of wireless energy transmission. The distance between box bottom to receiver’s wire loop is set 4 mm or 6 mm, respectively.

Subsequently, a 10 μm layer of positive photoresist (AZ 1512, Microchemicals GmbH, D-89079 Ulm, Germany) was applied on the titanium layer. The photoresist layer was irradiated by UV light through a mask whereby a wire pattern was created, as shown in Figure 1D. The irradiated areas of the photoresist layer were removed by a developer solution (AZ 300-MIF, Microchemicals GmbH, D-89079 Ulm, Germany). The remaining areas of the photoresist layer selectively masked the areas where electrode wires were obtained in the further process. In the etching process, the titanium layer was removed by wet etch, 50:1 dilute hydrofluoric acid (HF) (Ashland Chemical, Wilmington, DE, USA), in the exposed areas. The platinum layer was removed by deionized (DI) water:HCl:HNO_3_ in 1:3:1 (*v*/*v*), in the exposed area. Then, the residual photoresist layer was removed with a liquid immersion, with 20 sec in acetone and 20 sec in isopropanol. The areas where titanium/platinum was removed exposed polyimide as the surface layer. The base polyimide surface layer was activated and partially removed by reactive ion etching (RIE) in all areas uncovered by electrode wires. The surface was treated in 100 mTorr, 85% O_2_, 15% CF_4_ for 2 min at 200 W and 20 °C.

The surface was subsequently treated by KOH-deimidization bath at 25 °C for 5 min with manual agitation of the carrier boat at least every 60 s, followed with cascade rinse bath for 60 s, in triplicate. Then, the surface was dried with filtered nitrogen, and further re-treated in an HCl-deimidization bath at 25 °C for 5 min with mild agitation, followed by an identical cascade rinse bath. Finally, an about 250 μm-thick top layer of a precursor solution was applied by spin coating and cured to polyimide (PI2611, HD MicroSystems, US), which was combined with base polyimide after curing.

### 2.3. Protection Box Design for Wire Loops and Controller IC and Prototyping

To design a protection box to compartmentalize the receiver wire loops and controller IC from body fluid invasion after device implantation in vivo, reliable metal and insulation materials are crucial. We selected 6AL-4V ELI medical-grade titanium bulk as the crude material for protection box manufacturing and designed it as a round shape to match the physiological implantation. The prototype was outsourced to INTAI^®^ Inc., Co. (Taichung, Taiwan) for manufacturing, which sophisticatedly lathed under computer numerical control in compliance with our design, as shown in the Results section. A high-voltage vacuum capacitor amid the receiver wire loops and the passive components that surround the microcontroller integrated circuit (IC) was manufactured with titanium and ceramic composite (P/N: 1034101 Rev A, Greatbatch Medical, Minneapolis, MN, USA) for the consideration of biocompatibility with the shape of the trans-center of the anode and cathode to conduct electricity (Figure S1). A medical-grade epoxy resin (LOCTITE^®^ M-21HP^TM^, R. S. Hughes Company, Inc., Sunnyvale, CA, USA.) was used as the insulating and adhesive material for both glass and titanium box packaging. Another titanium box under the receiver wire loops was considered for the protection of the controller IC, which was designed as an irregular shape in compliance with the round shape of the protection box and fixed on the bottom titanium plate with three pieces of M2 screw. The flexible polyimide-based circuits were inserted into the bottom titanium plate to connect with the controller IC, the empty slot was filled with LOCTITE^®^ resin for a complete insulation, and overall, the titanium pieces were welded together to ensure complete packing.

## 3. Results

### 3.1. Architecture Design and Simulation of Hexahedron Shaped Wireless Energy Transmission

Firstly, we designed a rectangular box with a cortex wire architecture for the wireless energy transmission device, featuring a glass plate as the antenna support and surface packing material. This architecture designed here was different from the patented conventional implantable medical devices and prevented the use of materials with high magnetic conduction coefficients applied in the implantable wireless energy transmission. This architecture would save manufacture and design costs and be more comfortable for application on human implantation. The neuron electrode designed in this study was intended to manage pain through stimulations on the spinal nerve directly. Therefore, the electrode powered by wireless energy transfer should be placed adjacent to the spine and implanted subcutaneously, which was intended to implant in the side loin area below the rib cage and just above the pelvis as illustrated (Figure 1A). To avoid the uncomfortable feeling after implantation, the hexahedral corners of the energy receiver case were designed as a round shape (Figure 1B). The wires plane was placed 2.5 mm beneath the receiver case cover, and the wireless power transmission box was put about 7.5 mm in depth subcutaneously (based on the average thickness of human skin epidermal tissue being approximately 7.5 mm, and in compliance with the guidance established by Medtronic, Inc. to develop the neural stimulator (≤10 mm). The interval distance between cortex wires in the transmitter and receiver was approximately 10 mm (Figure 1C). This wireless energy transmission device was simulated to operate at the frequency of 13.56 MHz, and the transmission efficiency reached 85.2% (Figure 1D). To examine whether the cortex wires in the receiver side could receive the largest portion of energy transferring from the transmitter side, the top transmitter side was set to provide a 13.56 MHz radiofrequency energy of 500 mW for stimulation. The cortex wires in the receiver side were attached to the bottom of the receiver’s box. Data revealed the cortex wires in the receiver side did receive the largest portion of energy, which was shown in the red part (Figure 1E). Compared to the current distribution in the surrounding metal part, the transferred energy was concentrated in the receiver’s cortex wires (Figure 1E). Another experiment was carried out to explore whether the distance (h) between the box bottom to receiver’s wire loop would interfere with the total transmission efficiency. The parameter of the distance h was set to 4 mm or 6 mm, respectively, and the total simulated transmission efficiency could reach approximately 82% (Figure 1F).

### 3.2. Explore the Wireless Power Transmission Efficiency Influenced by the Skin Tissue Thickness

**Figure 2:**
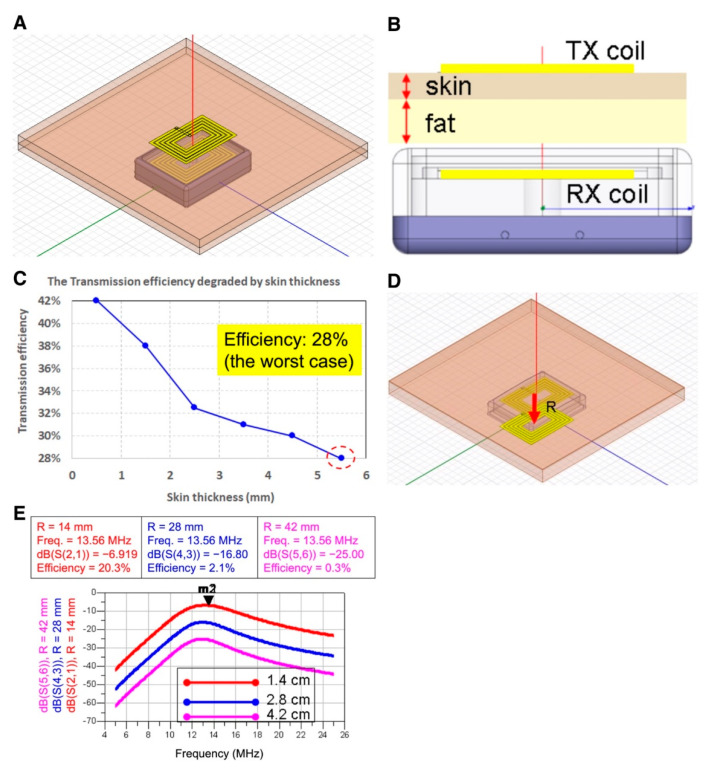
Effects of the skin tissue thickness and the misalignment of the cortex wire on the wireless energy transmission efficiency. (**A**) Skin tissue is placed in the simulation model. (**B**) Transversal view of skin tissue placed between the cortex wires in the transmitter and receiver. (**C**) Transmission efficiency is influenced by the thickness of skin tissue. (**D**) Analysis illustration of the cortex wire’s misalignment. R indicates the distance between the transmitter core and receiver core. (E) Simulation analysis of misalignment. R-values are 1.4, 2.8, or 4.2 cm separately.

To further explore the transmission efficiency of the hexahedron-shaped wireless energy transmission device influenced by the thickness of skin tissues, a 10 mm thickness of skin tissue was placed in the simulated model (Figure 2A) in which the receiver and transmitter coils were aligned precisely. According to Prof. Gabriel’s publications [12,13], the dielectric properties of human skin and fat tissues were used for electromagnetic simulation, that is, the fat tissue thickness was fixed at 5 mm and skin tissue thickness varied from 0.4 mm to 5.5 mm (Figure 2B). Due to the skin tissue possessing higher electrical conductivity, data revealed that the overall transmission efficiency remained at least 28% in the scenarios of skin thickness varying from the thinnest 0.4 mm (representing skin thickness of the eyelid) to the thickest 5.5 mm (representing the thickness of the plantar skin) (Figure 2C). The transmitter frequency was set at 13.56 MHz and 500 mW in the simulation process, and at least 100 mW at the receiver was achieved. The power provided by the wireless energy transmission system was very sufficient for many types of socket-on-chip (SoC) for neuron stimulation. Except for the influence of tissue thickness on the total transmission efficiency, the misalignment of the cortex wire was another critical factor that influenced the transmission efficiency. Addressing misalignment, we fixed the position of the receiver coils, where the center was circled by the transmitter coils. The distance between the two coil centers was termed as “R”, which was used as a factor to analyze the total transmission efficiency (Figure 2D). Data revealed the transmission efficiency was about 20% at R = 14 mm, and the transmission efficiency was only about 2% at R = 28 mm, respectively (Figure 2E). In the condition of coils misaligned, R = 28 mm and transmitter power = 500 mW, in which only 9.9 mW at the receiver was generated, which was very close to the critical threshold value of power for the SoC we designed for neural stimulation. Therefore, we recommended the misalignment distance for the user to operate neuron electrical stimulation should be set 28 mm at maximum.

### 3.3. Designs of Resonant Coupling Architecture for Wireless Energy Transmission

**Figure 3:**
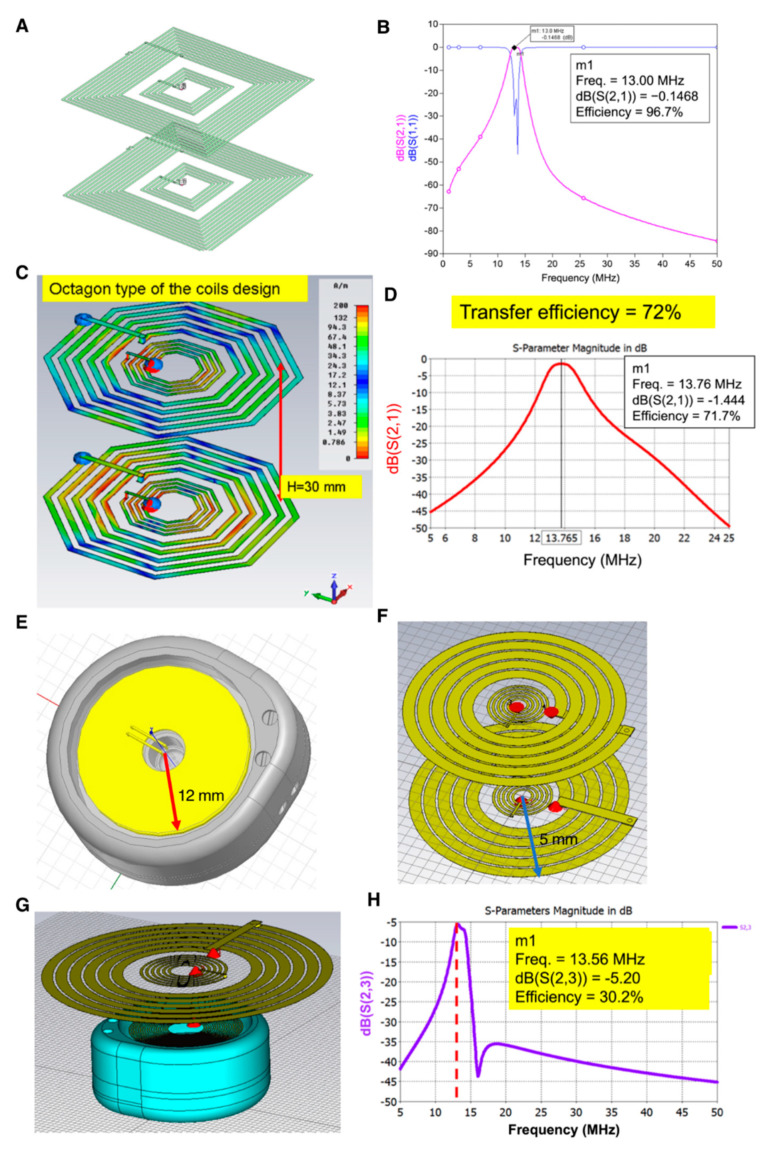
Resonant coupling architecture for wireless energy transmission. (**A**) Simulation model of resonant coupling in square cortex wires. (**B**) The transmission efficiency of resonant coupling in square cortex wires. (**C**) Design of round-shaped architecture with octahedral cortex wires. (**D**) The transmission efficiency of octahedral wires with resonant coupling technology. (**E**) Design of round-shaped titanium case for wireless energy transmission. (**F**) The Archimedes coils design in receiver. (**G**) Top to bottom view of round-shaped transmitter and receiver. (**H**) The transmission efficiency of the round-shaped circular wires with resonant coupling technology for wireless energy transmission.

To increase the transmission efficiency in a smaller transmitter and receiver area, we designed another architecture based on the resonant coupling principle as illustrated (Figure 3A). While the area of the cortex wires was designed in a 4 × 4 cm square and the operating frequency was set to 13 MHz, the eventual transmission efficiency reached 97% (Figure 3B). To ameliorate the uncomfortable feeling after wireless energy transmission device implantation, the device box manufactured using titanium was further designed with a round-shaped architecture with an octahedral high performance of the cortex wires (Figure 3C). The maximum transmission efficiency could reach 72% with the maximum radius being 1.75 cm after fine-tuned cortex wire electrical impedance (Figure 3D). For the titanium box, the inner diameter was designed in 24 mm as illustrated (Figure 3E), and the yellow part represents the cover of the octahedral cortex wires. In consideration of the close contact of the octagonal angles of cortex wires with the round-shaped titanium box to result in higher energy loss during electromagnetic transmission, we modified the cortex wire architecture in compliance with the Archimedes coils design to match with the titanium round-shaped box (Figure 3F). Besides, the cortex wires in the transmitter were also modified as the Archimedes coils design (Figure 3G). The outer radius of the transmitter was designed as 15 mm to reach an overall 30% transmission efficiency and a power of 120 mW, which would be highly sufficient for many SoC devices for neural stimulation (Figure 3H). Finally, we hand-made a receiver coil connected to a LED chip representing the SoC (Figure S2A), which received the power transferring wirelessly and lightened the LED (Figure S2B).

### 3.4. Designs of Neuron Stimulatory Electrodes

**Figure 4:**
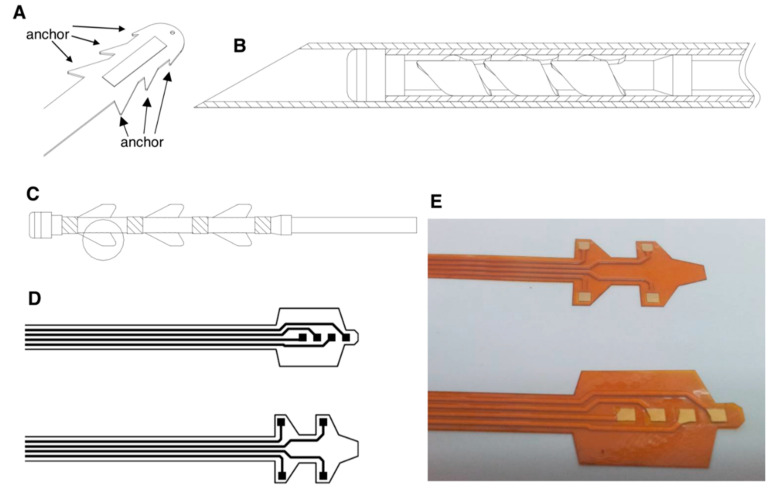
Designs of the neuron stimulatory electrodes. (**A**) The stretchable electrode is designed with fixing anchors on the edge. (**B**) Illustration of the guiding needle for the delivery of a stretchable electrode. (**C**) Expanded electrode with fixing anchors. (**D**) Deployment of an electrode on the stretchable matrix. (**E**) Two prototypes of stretchable electrodes made by polyimide.

To minimize the size of neuron stimulatory electrode and anchor the electrode adjacent to the dorsal root ganglion that indeed needs therapy, we developed a biocompatible and flexible electrode with fixing anchors available for microsurgery implantation. In our design principle, the flexible electrode would be protected and carried by a guiding hard needle to penetrate the spine dural space and spinal meninges (illustrated in Figure S3A), then to approach the soft tissues in the spinal cord, and finally anchor on the ganglion that needs electrical stimulation (illustrated in Figure S3B). Therefore, we designed the electrode with fixing anchors (Figure 4A), which would be rolled and inserted into the guiding needle for delivery (Figure 4B) and penetrate to reach the ganglion. When the electrode was positioned in the target site, the guiding needle would be withdrawn to expand the anchors to tightly insert itself into the diseased spinal ganglion (Figure 4C). The electrode was designed to precisely block the neural conduction pathway for pain signals transmission. Furthermore, the flexible electrode was designed in both architectures (Figure 4D), one with centralized stimulation microelectrodes aligned on the polyimide basement, and the other type with microelectrodes attached on the fixing anchors. The prototype of both flexible electrodes was manufactured by biocompatible material polyimide (Figure 4E).

### 3.5. Protection Box Prototype Manufactured for the Implantable Wireless Power Transmission System

**Figure 5:**
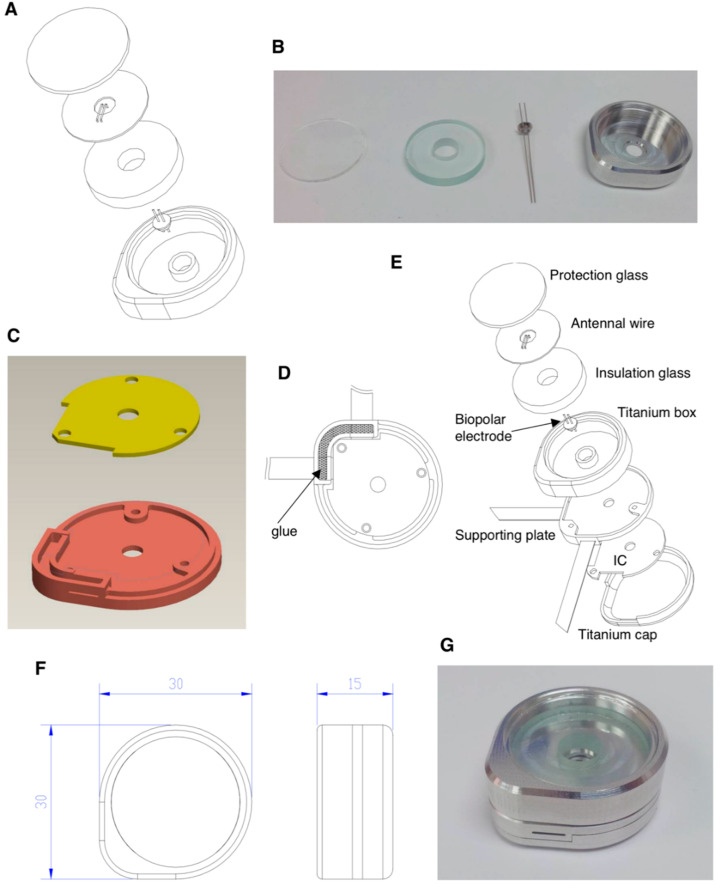
Scheme of the protection box for implantable wireless energy transmission system. (**A**) Scheme of the partial protection box for antennal wire in receiver side. (**B**) Prototype entities including the protection glass, insulation glass, bipolar electrode and titanium box. (**C**) Scheme of supporting titanium plate for controller IC protection. (**D**) Attachment of the stretchable electrode to the controller IC part by filling biocompatible glue, illustrated as black mesh. (**E**) Scheme of whole separated components. (**F**) Dimensions of the assembled protection box. (**G**) A complete assembly prototype of the protection box.

To design the protection box for the wireless power transmission system, we selected 6Al-4V ELI medical-grade titanium for the prototype manufacture in compliance with the guidance of ASTM F136-08 e1, ISO 5832.3-96, and BS 7572-3:1997. The medical-grade titanium was certificated by ultrasonic testing (UT) and recognized as AMS 2631B GR.1 CLASS AA. We first manufactured the protection box for cortex wires used on the receiver side in the wireless power transmission system as described in Materials and Methods, Section 2.3. The inner radius of the receiver box was 12 mm, and the distance between the cortex wire and receiver box inner wall should be more than 4 mm to ensure the transferred energy will be received completely. The receiver cortex wire was placed between two glass plates which provided satisfying insulation. The installation details were illustrated (Figure 5A), and the prototype of wire box was manufactured by titanium (Figure 5B). The controller IC which administrated the antennal wire to receive the transferred energy and the neural electrode to release the stimulatory energy was installed in the main protection case. Therefore, the controller IC-loaded protection box should be not only biocompatible but also more importantly, the complete biofluid insulation. We designed a titanium plate to load the controller IC, represented as yellow and an irregular part (Figure 5C), and to be fixed by three screw holes. The flexible electrode would be penetrated through the supporting titanium plate, which would be filled with biocompatible glue to attach the electrode to the microcontroller IC (Figure 5D). The overall components and the practical size were detailly schemed (Figure 5E,F). The titanium protection box prototype was manufactured and packaged sophisticatedly (Figure 5G).

## 4. Discussion

Electrical stimulation or heated energy was generally used to act on neurons in the spinal cord to block or interfere pain signaling to the brain. The pain signal transmission was generally prevented by the conventional continuous radiofrequency (CRF), which would generate a high temperature reaching 70–85 °C on the electrode tip to destroy the neural transmission and achieve pain relief [14,15,16,17].

Our PRF electrode designing concept is based on the spinal cord tissue structure, spread with many types of sensory neurons which are responsible for pain feeling transmission. The conventional therapy of administrating bulk and continuous stimulations on large areas of the spinal cord, including the dorsal root ganglion neurons, would be accompanied by significant side effects, such as paralysis, contraction, and a slightly uncomfortable feeling. Therefore, we aimed to design a tiny, stretchable and sophisticated neural stimulatory electrode possessing the capability to precisely fix adjacent to the dorsal root ganglion. Here, we explain the design concept and show the hands-on prototype.

Our first design of a wireless power transmission system possessed at least 80% transmission efficiency, providing very sufficient power for a general system-on-chip (SoC) to work in a common circumstance [18] while working with a low power 0.5 W radiofrequency transmitter. The power generated from this wireless energy transmission produced at least 300 mW for neuron stimulation (Figure 1), and fulfilled the requirement for the misalignment of receiver and transmitter (Figure 2), making this wireless energy transmission design more robust and practical.

In terms of the wireless energy transmission efficiency influenced by skin thickness, simulation results indicated that overall energy transmission efficiency maintained >28% at 5.5 mm skin thickness setting, which is approximately equal to footpad skin thickness. While the transmitter was set at 13.56 MHz, 0.5 W, overall transmission efficiency could reach ≥100 mW, which was sufficient for the power supply for most of the neuron stimulatory SoC (Figure 2). In the misalignment simulation of transmitter and receiver wire loops, the overall transmission efficiency decreased to 9.9 mW in the circumstance of 0.5 W transmitter power, and 28 mm misalignment in both wire loop centers, approaching the minimum requirement of power supply for SoC in our other studies (data not shown). Therefore, the largest misalignment of receiver and transmitter should be set ≤28 mm for therapy.

Skin thickness apparently interferes with the transmission efficiency of the wireless power transfer. The conductivity of physiological tissue increased with the blood content in tissues, resulting in diminished wireless power transmission efficiency [19]. Therefore, we intended to utilize a resonant wire loop design for the wireless power transfer and termed as the “second wire loop” design, which increased the transmission distance in the identical wire loop area. In the “first wire loop” design, when the distance between transmitter and receiver was 10 mm, the transmission efficiency could reach 80%. However, when the distance increased to 30 mm, the transmission efficiency dropped significantly to be 20%. However, in the resonant wire loop design (i.e., second wire loop design), the transmission efficiency of an identical wire loop area 4 × 4 cm as the first wire loop design could reach 97% in an operation frequency set to 13 MHz (Figure 3).

The transmission efficiency of the resonant wire loop designed in an octahedral shape holds superior transmission efficiency than that of the rectangular shape. However, the angle of the octahedral wire loop would be very close to the inner wall of the receiver case manufactured in titanium, leading to significant energy loss on the receiver side. Therefore, we modified the wire loop design to a round shape in the second wire loop design, in which the radius of the receiver and transmitter wire loop was 12 mm and 15 mm [20], respectively (Figure 3). The overall transmission efficiency reached 30%, providing 120 mW power transfer which is sufficient for most SoC power requirements [18,21]. In this study, the development of wireless power transmission for implantable neuron electrode power supply has finished the fine-tuned wire loop design simulation and efficiency evaluation, while the resonant wire loop module design provided longer distance energy transmission and higher efficiency, reaching a maximum of 30 mm in the distance and 96% in efficiency, respectively. Therefore, by applying the wireless power transmission design to the field of integrated implantable medical devices, such as neuron stimulatory electrodes, pain administrable chips, or retinal prosthesis, the recipient could avoid the drawbacks of repeated devices recharging [22].

The physical locations of pain sensory neurons are distinct between individual patients. Therefore, the neurosurgeons examine the exact sensory neuron location assisted by an in vitro monitor system and patients’ self-feeling, and decide the sites that need electrode stimuli. Due to the conventional electrode design without a fixing anchor on the electrode tip, the stimulus would affect a large area accompanied with significant inevitable side effects [14,15,16,17]. When the therapeutic effects diminished, doctors generally increased the stimulatory dose to compensate for the lower therapeutic efficacy, which would increase the risk of safety concerns. Therefore, this study aimed to design a PRF electrode possessing the capability to precisely administer the stimuli on the spinal cord ganglion neurons which need therapeutic stimuli (the overall architecture is illustrated in Figure S3A).

The manufacturing features of the neuron stimulatory electrode must possess not only biocompatibility for the recipient, but also bending flexibility for surgeons to conduct micro-surgical implantation conveniently and reduce the consequent complications. The electrode materials are conventionally manufactured with silicon rubber or polydimethylsiloxane (PDMS) and coated with reliable metals such as gold, silver or platinum, to serve as conducting wires to fulfill the requirement for biocompatibility and flexibility.

When aiming for life-long implantation of the pain-administrable device in the human body, it is inevitable that a metallic box will be used to protect the controller IC, which administrates wire loops to receive energy wirelessly, meanwhile releasing energy to the electrode for neural stimulation. Besides, the metal shielded effect retards the wireless energy transfer, and the electrode should be placed adjacent to the ganglion which needs stimulation. Both antenna wire loops and electrodes should be installed and connected to the outer surface of the metallic protection box (the implantation scenario is illustrated in Figure S4B). Therefore, reliable metallic material and excellent insulation, such as titanium and silicone medical adhesive, all fulfilling the requirement of biocompatibility should be considered (Figure 5). In terms of experimental prototyping, we selected the resonant coupling architecture design as illustrated (Figure 3G) for prototype manufacture. The prototype was composed of multi resonant wire loops and provided long-distance transmission efficiency, reaching 30 mm in maximum and ≥96% transmission efficiency, which was sufficient for lots of implanted medical applications.

To avoid the metallic box interfering with the energy transmission, the metallic box bottom should be separated from receiver wire loops ≥4 mm using thick glass plate protection (Figure 5A), and top-covered with a thin glass plate. The energy received from antenna wire loops is transferred to the microcontroller IC through the high-voltage vacuum capacitor (Figure S1) with a medical-grade titanium and ceramic composite materials manufactured anode and cathode, which provides biocompatibility. Besides, the insulation package is designed to protect the internal electronic chips of the implantable PRF neuron stimulatory electrode from water, body fluid, vapor, chemical invasion, and mechanical force loading; meanwhile, it also functions to prevent leaking out of any biohazard component in the electronic chips. The medical-grade epoxy resin is considered for the insulating and adhesive material for both glass and titanium box packaging, which is expected to provide a biocompatible water-proof capability. Furthermore, the design and material of the insulation package of an implantable medical device are crucial when considering the biocompatibility. Another critical consideration for the PRF neural electrode is the wireless power transmission and signal-controlled module, which receives the power and control signals transmitted by wire loop magnetic coupling constructed in the outside part to achieve the wireless energy transmission. Our study intended to finetune the design of the wire loop to optimize the transmission efficiency through simulation and prototype manufacturing. Collectively, our study designed an implantable PRF neuron stimulatory device with a titanium box package and excellent biocompatible glue to provide a water-proof capability.

## 5. Conclusions

We have demonstrated the critical criteria to manufacture an excellent implantable PRF device, including (1) efficient transdermal wireless power transferred architecture, providing longer distance energy transmission (30 mm) and higher efficiency (≥96%). Our architecture provided 120 mW wireless power transfer, which was sufficient for most of the SoC power requirements and benefits the recipient as they avoid repeated surgery for device recharging. (2) A fixable and stretchable PRF stimulatory electrode, providing precise stimulation and surgical flexibility in compliance with the operation conducted in the microsurgical implantation, and ameliorating the complications elicited by PRF implantation. (3) Titanium box packaging and insulation design, possessing good biocompatibility in vivo. Overall, we proposed a feasible PRF neural stimulatory device with efficient wireless energy transmission, which could be further applied to the development of other implanted systems and emerge as a critical technology platform for neural electrodes.

## Data Availability

Data is contained within the article or supplementary material.

## Supplementary Materials

The following are available online at https://www.mdpi.com/2072-666X/12/4/356/s1, Figure S1: Trans-center anode and cathode used to conduct electricity, S2: Prototype of the hand-made receiver coil, S3: Dorsal root ganglion and stimulatory illustration, S4: Over wireless power transmission and electrode design, and implantation illustration.
