# Supplementary material for: Pain-Administrable Neuron Electrode with Wireless Energy Transmission: Architecture Design and Prototyping
Source: Micromachines (Basel). 2021 Mar 25;12(4):356. doi: 10.3390/mi12040356 (PMC8065446; doi:10.3390/mi12040356)
Supplement: Supplementary file 1 [file micromachines-12-00356-s001.pdf]

micromachines

MDPI

Article

Pain-Administrable Neuron Electrode with Wireless Energy Transmission: Architecture Design and Prototyping

Chin-Yu Lin [1,2,*], Li-Chi Chang [3], Jyh-Chern Chen [4,*], Meng-Sheng Chen [5], Hsun Yu [5] and Mei-Chih Wang [6,*]

[1] Institute of New Drug Development, China Medical University, Taichung, 40402, Taiwan
[2] Master Program for Biomedical Engineering, Collage of Biomedical Engineering, China Medical University, Taichung, 40402, Taiwan
[3] Graduate Institute of Communication Engineering, National Taiwan University, Taipei City 10617, Taiwan; D02942003@ntu.edu.tw
[4] ACE Biotek Co., Ltd., Hsinchu, 30261, Taiwan
[5] Electronic & Optoelectronic System Research Laboratories, Industrial Technology Research Institute, Hsinchu 31057, Taiwan; mason@itri.org.tw (M.-S.C.); syunyu@itri.org.tw (H.Y.)
[6] Biomedical Technology & Device Research Laboratories, Industrial Technology Research Institute, Hsinchu, 31057, Taiwan
* Correspondence: geant@mail.cmu.edu.tw (C.-Y.L.); jc_chen@acebiotek.com (J.-C.C.); mcwang@itri.org.tw (M.-C.W.); Tel.: +886-4-2205-3366 ext. 8108 (C.-Y.L.); +886-3-5912812 (M.-C. W.); +886-3-5500909 (ext. 3402) (J.-C. C.)

Citation: Lin, C.-Y.; Chang, L.-C.; Chen, J.-C.; Chen, M.-S.; Yu, H.; Wang, M.-C. Pain-Administrable Neuron Electrode with Wireless Energy Transmission: Architecture Design and Prototyping. *Micromachines* **2021**, *12*, 356. https://doi.org/10.3390/mi12040356

Academic Editor: Beelee Chua

Received: 10 February 2021
Accepted: 22 March 2021
Published: 25 March 2021

Publisher's Note: MDPI stays neutral with regard to jurisdictional claims in published maps and institutional affiliations.

Copyright: © 2021 by the authors. Licensee MDPI, Basel, Switzerland. This article is an open access article distributed under the terms and conditions of the Creative Commons Attribution (CC BY) license (http://creativecommons.org/licenses/by/4.0/).

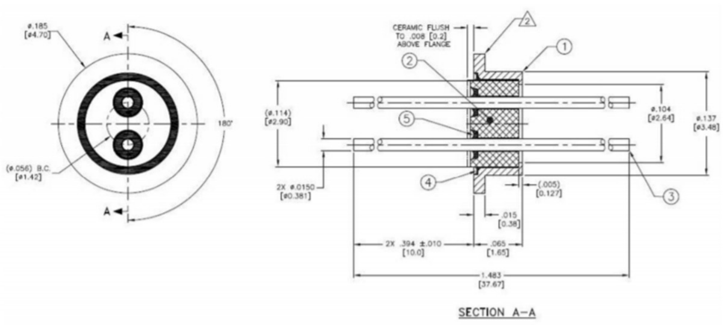

Figure S1. Trans-centre anode and cathode used to conduct electricity. A high-voltage vacuum capacitor amid at receiver wire loops and the passive components surround the microcontroller IC was manufactured by titanium and ceramic composite (P/N: 1034101 Rev A, Greatbatch Medical, US) for the consideration of biocompatibility with the shape of trans-centre of anode and cathode to conduct electricity.

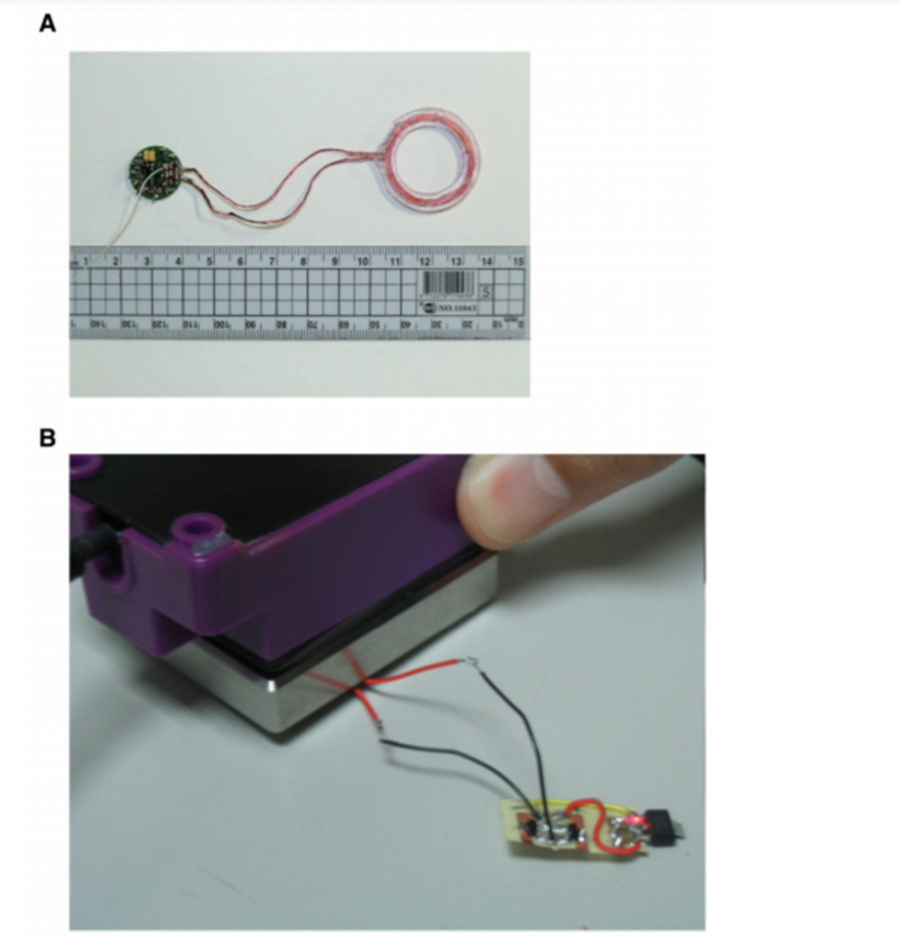

Figure S2. Prototype of hand-made receiver coil. (**A**) A hand-made receiver coil connected to a LED chip representing the SOC. (**B**) The LED was lightened when received the power from wireless power transmission.

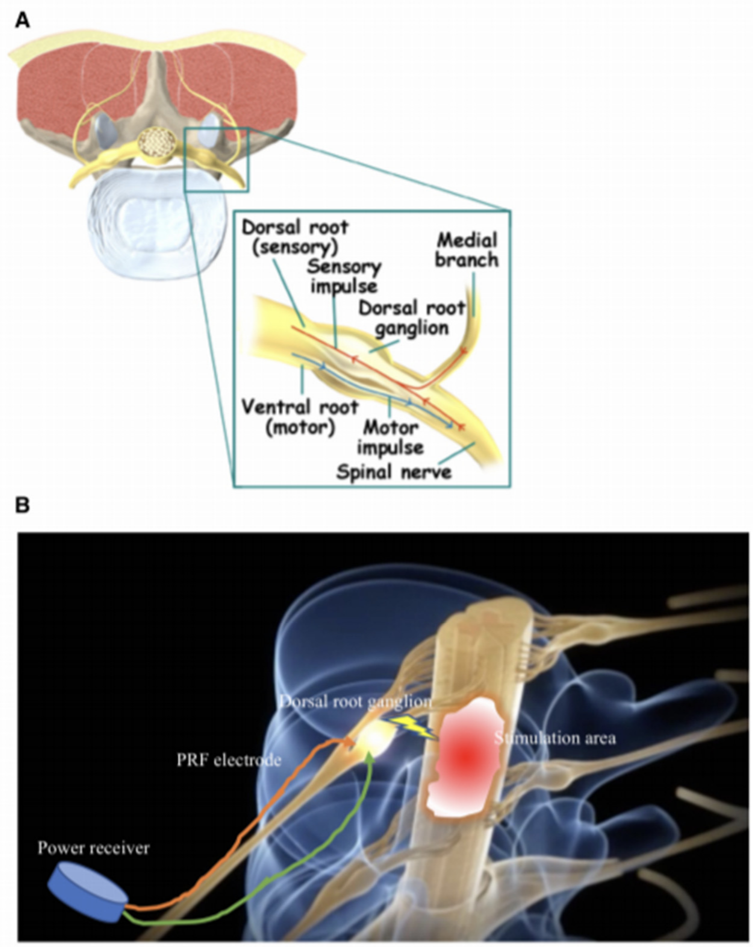

Figure S3. Sensory dorsal root and the dorsal root ganglion (DRG) stimulatory illustration. (**A**) Stimulatory impulses of sensory dorsal and motor ventral roots in the spinal nerve are demonstrated. (**B**) The flexible electrode is firstly protected and carried by a guiding hard needle to penetrate the spine dural space and spinal meninges. Secondly, the guiding needle is withdrawn and the flexible electrode will stretch out and anchor on the DRG to elicit the stimulation.

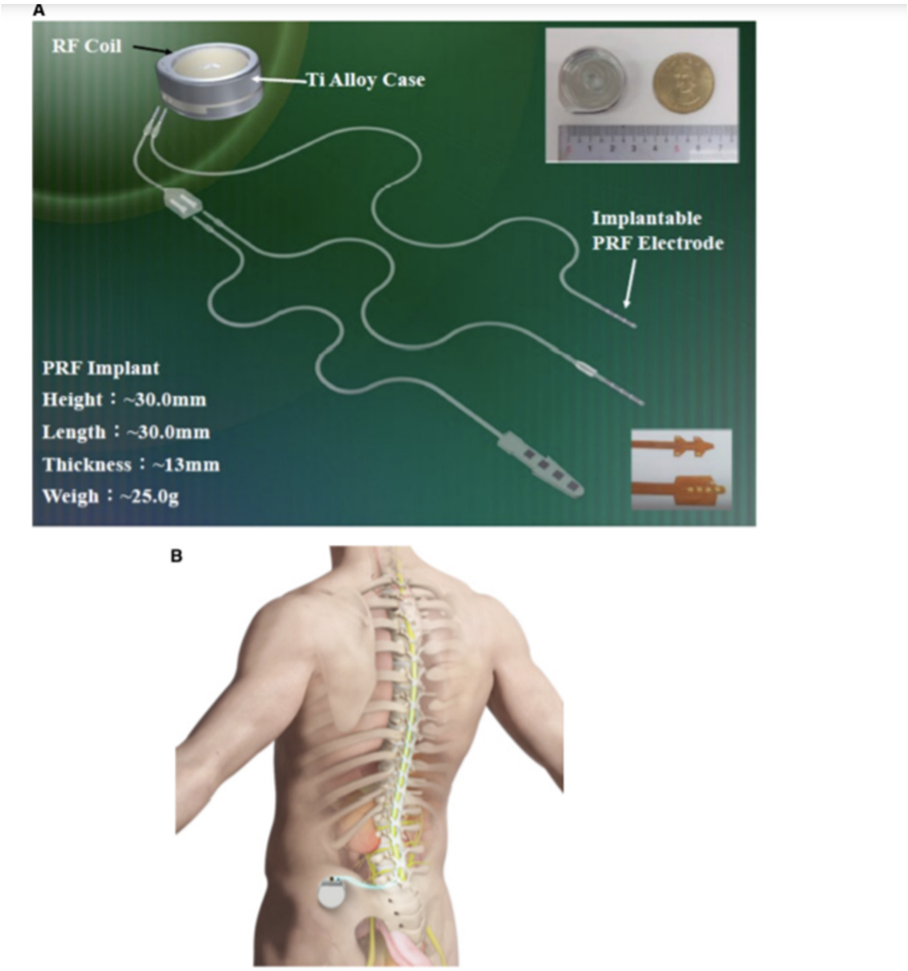

Figure S4. PRF wireless power transmission receiver, three electrode prototype design and implantation illustration. (**A**) Architecture design of PRF neuron stimulatory electrode and wireless power transmission system. (**B**) Human body implantation scenario. .
